# Inertial Sensor-Based Gait Recognition: A Review

**DOI:** 10.3390/s150922089

**Published:** 2015-09-02

**Authors:** Sebastijan Sprager, Matjaz B. Juric

**Affiliations:** Faculty of Computer and Information Science, University of Ljubljana, Vecna pot 113, SI-1000 Ljubljana, Slovenia; E-Mail: matjaz.juric@fri.uni-lj.si

**Keywords:** inertial sensors, inertial data, gait analysis, gait recognition, gait identification, gait authentication, biometry, gait patterns, review

## Abstract

With the recent development of microelectromechanical systems (MEMS), inertial sensors have become widely used in the research of wearable gait analysis due to several factors, such as being easy-to-use and low-cost. Considering the fact that each individual has a unique way of walking, inertial sensors can be applied to the problem of gait recognition where assessed gait can be interpreted as a biometric trait. Thus, inertial sensor-based gait recognition has a great potential to play an important role in many security-related applications. Since inertial sensors are included in smart devices that are nowadays present at every step, inertial sensor-based gait recognition has become very attractive and emerging field of research that has provided many interesting discoveries recently. This paper provides a thorough and systematic review of current state-of-the-art in this field of research. Review procedure has revealed that the latest advanced inertial sensor-based gait recognition approaches are able to sufficiently recognise the users when relying on inertial data obtained during gait by single commercially available smart device in controlled circumstances, including fixed placement and small variations in gait. Furthermore, these approaches have also revealed considerable breakthrough by realistic use in uncontrolled circumstances, showing great potential for their further development and wide applicability.

## 1. Introduction

Rapid development of microelectromechanical systems (MEMS) has paved the way to some significant scientific and applicable breakthroughs in several research areas. Undoubtedly, one of the most important members of MEMS family are inertial sensors (accelerometers, gyroscopes) that are commonly combined together as inertial measurement units (IMU). Due to many positive characteristics, such as lightweight, small-size, low power consumption, portability and low-cost, inertial sensors have become widely used and indispensable in all activities that indirectly or directly addresses motion. Furthermore, it has long been known that data acquired by inertial sensors can be processed by advanced approaches in order to perform complex motion analysis. Particular attention is devoted to the problem of gait analysis since walking ability represents one of the most important vital functions that has significant influence on the quality of life.

Nowadays, performing gait analysis with inertial sensors as an important group of wearable sensors [1,2] has become indispensable in several fields of research including biomechanics, neurorehabilitation, sport medicine, *etc.* [3,4,5,6,7]. Inertial sensors can measure single or multi-point motion trajectories of single or multiple body segments of the subject during gait. During the measurement period, uni- or multivariate signals are acquired that provide instantaneous information on measured quantity (*i.e.*, spatial accelerations when using triaxial accelerometer). In this manner, subject’s gait can be assessed in terms of gait parameters that can be interpreted in several ways in order to discover or to observe specific phenomenon, including inter- and intra-subject assessment of gait variations based on gait pattern similarity. On the other hand, considering the fact that each individual possesses a unique manner of walking, gait assessment relying on inertial sensors can be in analogous way exploited for the problem of gait-based recognition. In this manner, gait can be interpreted as a biometric trait and, consequently, inertial sensors have great potential to play an important role in the field of biometry. Thus, the application of such biometric approach can significantly strengthen security aspects that can be represented by several use-case scenarios, including a novel verification procedure that can extend or even replace existing security mechanisms (*i.e.*, more convenient that manual entry of PIN number), theft detection, profile switching, user tracking, support to mobile healthcare systems, and many others.

Furthermore, it should be mentioned that the development of inertial sensor-based gait recognition approaches emerged simultaneously with the wide occurrence of ubiquitous smart devices, especially smartphones and tablets. Nowadays, integration of inertial sensors in smart devices has become a standard. There are two crucial facts that expose the applicability of inertial sensors as an important part of ubiquitous smart devices in terms of gait analysis. First, there is a large pool of potential users that possess, carry and use smart devices on a daily basis. In 2012, In-Stat reported that by end of 2015, 65% of the U.S. population will own a tablet or a smartphone having inertial sensors integrated [8]. In fact, it can be assumed that actual number will overreach these expectations. Second, inertial sensors as a part of smart devices are powerful tool and are not longer strictly limited to support simple and trivial task only (*i.e.*, tilt estimation) as it was primarily intended at their appearance due to several limitations (energy efficiency, computational power, data transfer bandwidth and cost, storage) that were partly or fully overcome recently with the latest achievements in the field of pervasive computing. In fact, it has already been shown that inertial data acquired by sensors in ubiquitous smart devices can be used in order to assess users’ motion in advanced manner, including localization as one of the most intriguing challenges recently, as well as activity recognition and advanced motion analysis including gait. Such approaches have been examined in several areas, mostly in sports (*i.e.*, step count and gait speed estimation [9]) and clinical applications (assessment of user’s health state based on gait abnormalities [10], fall detection [11], *etc.*). Thus, the assumption that gait recognition relying on inertial data acquired by using ubiquitous smart devices has become reasonable and has been addressed by many research groups recently.

Nowadays, with appearance of ubiquitous devices, including sensors, smart devices (phones, tablets) as well as small and wearable single-board computing systems, pervasive computing has become indispensable in this context. One of the most important aspects of pervasive computing is interconnectivity and interoperability of ubiquitous devices. Such concept is also known as Internet of Things (IoT) with main purpose of integrating intelligent devices, technologies on several levels, including data, communication, decision-making and application level [12,13]. IoT goes hand-in-hand with cloud computing, represented as the computation paradigm of the future. In this manner, connectivity is the most important issue that needs to be resolved. However, capabilities of latest ubiquitous devices, including their performance and autonomy, ensure efficient data processing and communication with cloud system that relies on the bandwidth and cost of data transfer. Thus, such paradigm is expected to become completely feasible since trends show that ratio bandwidth-cost is growing significantly. In such way, one can benefit from the following: efficient large data-stream transmission, heavy computational processing on server side, reasonable latency and large data store. Considering these factors, inertial sensors as a significant part of ubiquitous devices should strengthen their role as they can be applied for more complex tasks that are performed continuously. In this context, the approaches that allow for advanced movement and gait analysis based on inertial data should be taken into careful consideration. This also includes gait recognition approaches that could become one of the most important mechanisms either in the field of biometry, security, telemedicine, biomedical engineering and many others.

According to these facts, one of the most intriguing research questions is whether it is possible to recognise (either identify or authenticate) user by the way he walks by using either single or multiple inertial sensors, and even more—Whether is the recognition applicable by smart devices (phones, tablets) that are widely accessible and used frequently. There has been made a significant progress in this field of research recently. However, at this time there is no review on a current status of inertial sensor-based gait recognition, which represents the main topic of this paper. Consequently, we believe that the time is right to provide a systematic review of methodologies that sufficiently cope with the problem of inertial sensor-based gait recognition and its findings. Thus, main purpose of this review paper is to meet the following objectives: to present current state-of-the-art on the field of inertial sensor-based gait recognition in systematic way, to evaluate and to compare recent approaches that have made the most significant contribution and, finally, to identify guidelines for the further development on this emerging and attractive field of research.

## 2. Review Process

**Figure 1:**
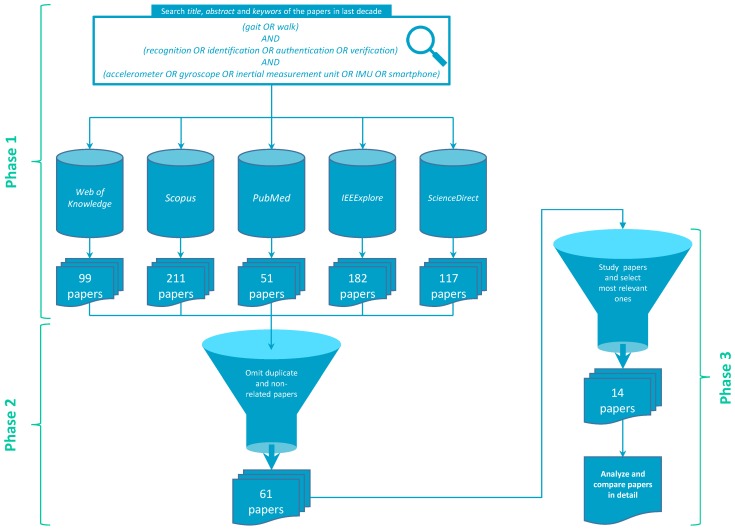
Detailed flowchart of a review process.

Whole review process was performed systematically—It was divided into three phases as shown in Figure 1. The structure of the paper strictly follows these directions. In the first phase, according to several review questions, a collection of papers is identified and analysed based on the keywords that correspond to these open questions. Second phase provides a systematic overview of the methodology generated from all significant approaches published in the last decade (Section 4). In third phase, thorough comparative analysis of recent and most significant contributions is provided (Section 5). Finally, based on findings discovered through the review process, current state-of-the-art is discussed in Section 6 while open possibilities and directions for further development are provided in Section 7.

Initial phase of review demanded a formation of research questions that need to be considered in order to fully cover the research area of inertial sensor-based gait recognition and to justify the applicability and advisability of its use. Therefore, the answers to the following seven questions are provided through this review paper relying on the proposed three-phased review process:

- How it is possible to recognise an user by the way he walks relying solely on data acquired by single inertial sensor, multiple inertial sensors or their fusion?
- What are the methodological constraints and how are they addressed?
- What are the physiological (gait-related) constraints and how are they addressed?
- How is the evaluation procedure performed and what is the relevance of the evaluation results?
- What is the performance and reliability of the most efficient approaches?
- What is the potential for the general use in realistic circumstances?
- What are the open problems and in which direction the further development is aimed?

In order to sufficiently identify all corresponding journal papers and proceedings, the following keywords were used to search for their appearance in the title, abstract and keywords of the papers: *(gait OR walk) AND (recognition OR identification OR authentication OR verification) AND (accelerometer OR gyroscope OR inertial measurement unit OR IMU OR smartphone)*. Basically, the searching phrase had to cover three aspects: *observed phenomenon* (gait or walk), *how the observed phenomenon is measured* (keywords directly related to inertial sensors) and *what we do with measured phenomenon* (recognition also covers identification, authentication of verification—Further details on this partition are provided later). Thus, in the first phase of the review process, these keywords were passed to search engines of the following databases and digital libraries (number of discovered papers for a particular database is provided in parentheses): Web of Knowledge (99), Scopus (211), PubMed (51), IEEEXplore (182) and ScienceDirect (117). The selection of these specific datasets stems from their significance in the field of engineering, as well as in biomechanics, medicine, biometry and security.

In second phase, from obtained 660 papers, the duplicates were removed in the first place. After the careful consideration of all abstracts, the papers that were insignificant or were not directly related to the problem of inertial sensor-based gait recognition were omitted. In this manner, 61 papers (about 10% of initial number) that fully cover the reviewed topic were obtained. These were checked in the light of above mentioned research questions in the similar way as proposed by Black and Downs in [14]. Selected papers were then studied thoroughly. Based on the findings, a systematic review and a methodological layout of inertial sensor-based gait recognition approach were generated.

After careful review of the papers performed in the second phase, 14 papers that reflect the most significant contribution on the reviewed topic were selected as the representatives in the third phase. Majority of these papers were published in recognised journals while some of them were published in proceedings of significant conferences. Papers selected in this pool had to provide answers to the majority of research questions stated above, where these answers had to be fully supported by methodological and experimental appropriate and relevant findings (*i.e.*, extensive experimental protocol, high performance supported by statistically significant evaluation procedure, *etc.*). On the foundations of these representative papers, thorough comparative analysis was performed as well as some interesting conclusions have been made.

## 3. Background

In general, gait patterns can be identified and analysed in two ways: they can be estimated explicitly as physiological gait parameters, *i.e.*, spatio-temporal parameters (cadence, step length, gait symmetry, inner-foot distance, *etc.*) [15], detection of gait phases [5] or kinematic parameters (joint angle measurement) [4,16] either expressed implicitly by applying special feature extraction techniques. Parameter-based analysis is crucial when observing the behaviour of individual parts of the locomotor apparatus. On the other hand, feature-based gait patterns do not provide exact physiological properties on movement in terms of locomotor apparatus since they rely on the data transformation which depends on the selected feature extraction techniques, but still implicitly hold the information on the movement.

The problem of gait recognition is primarily focused on natural gait. It can be defined as straight walking sequence of the individual, where the expected step frequency is constrained by interval with lower limit between 0.7 and 0.75 Hz and upper limit between 1.35 and 1.51 Hz as reported in [17,18]. However, it is very important to consider that natural gait can be easily perturbed by several gait-affecting factors, that can lead into minor or even drastic alterations of individual’s gait patterns. In general, these factors can be divided in the following two groups: (a) physiological and (b) environmental factors. From the individual’s point of view, physiological factors can induce gait pattern alterations based on two modes: either regulated automatically (unconscious) or by conscious control (*i.e.*, deliberate changing the manner of walking). Factors related to the unconscious mode can be further divided into two subgroups: permanent (*i.e.*, chronic gait abnormalities), and temporal varying/occasional (*i.e.*, acute gait abnormalities, mood, age-related factors, *etc.*). Environmental factors are represented by all external entities that directly or indirectly cause the variations in gait patterns of the individuals—These are mostly related to clothing, footwear, walking surfaces, slope and obstacles.

Based on these facts, we can conclude that gait analysis represents a complex process applied by advanced approaches that are based on peer-comparison of gait patterns of individuals determined by several gait parameters with proper consideration of gait-affecting factors. In the context of gait recognition it is sufficient if similarity of individuals’ gait patterns is assessed and compared in a proper way, since it is stated as the problem of the observation of gait patterns that will be similar for the observed subject and discriminatory enough to distinguish them from the patterns of all other subjects (inter-subject similarity of gait patterns). Additionally, when the effects of gait-affecting factors are considered, the observation of intra-subject gait pattern similarity for each factor takes place.

The idea to carry out sensor-based gait recognition extends over more than 30 years ago [19], when first attempts performed by observation of motion trajectories obtained from light sources mounted on joints have shown feasibility of such approach. Consequentially, hand-in-hand with the rapid development of sensing technology, new gait recognition approaches have appeared recently. They can be divided into (a) video-based [20], (b) floor-based [21] and (c) wearable sensor-based gait recognition approaches [22]. The latter have drawn particular attention in this field of research with the development of MEMS inertial sensors, especially due to their positive characteristics as presented in previous section. The development of inertial sensor-based gait recognition approaches has occurred in last decade. At this point, the contributions from the research groups of Ailisto and Mantyjarvi [23,24] as well as Gafurov [25] for their pioneer research activities on this field of research should be emphasised. The development significantly emerged in last 5 years with the wide appearance of smart devices, whereas further development is expected in the near future (see Section 7 for more details).

By introducing gait as a biometric trait that can be employed using inertial sensors, two different identity management functionalities should be determined as described in [26]. Thus, gait recognition can be performed as an identification, where subjects tries to identify himself without providing its identity explicitly and the approach tries to identify the subject. On the other side, by the authentication (or verification often used as its synonym) subject claims an identity and the biometric approach verifies if the claim is genuine. As all biometric approaches, gait recognition operates in two phases: enrolment phase and recognition phase. By enrolment phase, subject provides his identity information and data acquired by inertial sensors during gait. Inertial data is transformed into gait patterns and stored in database along with subject’s information on identity. As already mentioned, recognition phase can be performed either as an identification or authentication phase. By identification phase, subject provides data acquired by inertial sensors during gait only at this time. Incoming gait pattern is then compared with gait patterns stored in database. Recognition procedure then determines whether input pattern can be matched with the stored ones and provides the information of subject’s identity if the subjects is genuine or if the subject is declared as impostor. By authentication phase, subject besides gait data also claims its identity. Recognition procedure then compares incoming gait pattern with the gait patterns stored in the database that correspond to the claimed identity. If compared patterns are similar, subject is identified sufficiently or is rejected otherwise.

## 4. Methodology—A General Overview

**Figure 2:**
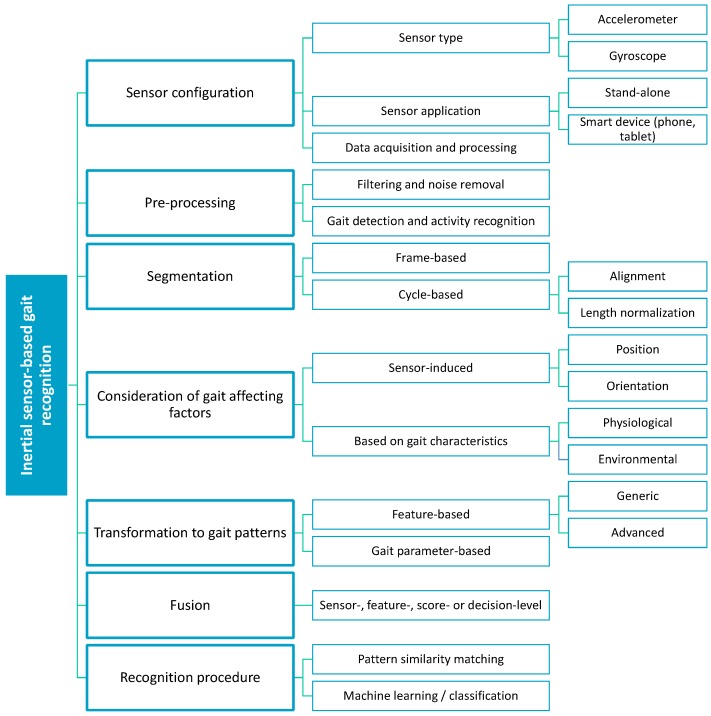
Methodological layout of existing inertial sensor-based gait recognition approaches.

The basic overview of existing inertial sensor-based gait recognition approaches is depicted in Figure 2 and described during the following subsections in more detail. In general, all approaches operate according to the following principle: (a) based on the appropriate sensor set-up, inertial data is acquired during user’s gait; (b) following pre-processing and segmentation step, acquired inertial data is transformed to gait patterns; (c) incoming gait patterns are compared with enrolled patterns by appropriate recognition procedure. During these phases, some approaches may also leverage fusion procedure. Last but not least, gait-affecting factors are also considered in some of the approaches.

### 4.1. Sensor Set-up and Data Acquisition

Inertial data during gait can be measured by using inertial sensors in two different configurations: either as stand-alone sensors (evaluation board) or sensors embedded in commercially available smart devices. The first ones are used for experimental purposes where three acquisition parameters can be configured: sampling frequency, measuring range and resolution. These should be considered due to the subsequent processing of acquired inertial signals in order to avoid unwanted distortion that can affect the shape of gait patterns (*i.e.*, aliasing, clipping or quantization error). Additionally, stand-alone sensors allow for experimenting with arbitrary sensor installation which can be applied in order to examine the influence of sensor-induced factors on recognition (*i.e.*, position, orientation) [27,28,29]. Furthermore, such configuration is also applicable as a supplementary part of body area network [30].

One of the most important research question was whether it is possible to replace special sensor configuration with commonly used and commercially available smart devices and to cope with the limitations that appear as a consequence of casual use. Sprager *et al.* [31] followed by many others [32,33,34] have reported first successful attempts of gait recognition based on the inertial data acquired by smartphones. Unlike as by stand-alone configuration, inertial sensors are integrated on a circuit board at various positions and stored inside smart devices depending on the different models of various manufacturers. For the purpose of collecting inertial data in a standardised way, acquisition parameters are controlled by sensor API, allowing application developers to pick sampling rate in indicative manner only. It is also desirable to ensure power efficiency and longer battery autonomy by sampling inertial data with the rate low as possible. Additionally, sample rates are usually time-varying, thus additional step should be performed in order to ensure equidistant sampling intervals for further processing. This is usually performed by interpolation, either linear or cubic [31,32,33,34,35,36,37,38,39]. Nevertheless, sampling rate must be sufficiently high in order to cover all dynamic changes that are induced in acquired inertial data during gait. Most of papers report that for natural gait it is enough to set the sampling rate in the range above a few tens of Hz. In the very first investigations, researchers experimented with relatively high sampling rates around 250 Hz [23,24,40,41]. In the following years, majority of stand-alone sensor-based approaches used the sampling rates in the range between 50 and 100 Hz [27,29,42,43]. Similarly, smartphone-based approaches relied on sampling rates below 100 Hz with most efficient approaches even using relatively low sample rate of 25 Hz [44,45,46].

Detailed specification of sensors used in the recent approaches that serve as representative studies in the review process are shown in Table 1. As already mentioned, inertial data during gait is usually acquired by two types of sensor: accelerometers and gyroscopes. As expected, majority of approaches relies on accelerometer data, since gait dynamics is well-reflected in measured accelerations. On the other side, there were same attempts to perform gait recognition based solely on angular velocities [42,45,47] resulting in lower recognition performance when compared with the accelerometer-based recognition. However, major contribution of gyroscopes is in their combination with accelerometers (as IMU) either applied to compensate orientation errors or to perform fused recognition, as explained in Section 4.7 in more detail. Typical measuring range of accelerations used for gait recognition is between ±2 g and ±8 g, while angular velocities measured by gyroscopes are usually in the range between $\pm 150^{\circ }$/s up to $\pm 2000^{\circ }$/s. Concerning reported resolutions, inertial data is digitalised by A/D converters typically within the interval from 8 to 12 bit.

Inertial data is collected either by (a) univariate, (b) multivariate or (c) multi-sensor signals. Univariate signals are used mostly by early smartphone-based gait recognition approaches as acceleration magnitude obtained by transformation of multivariate acceleration measurements. This representation is popular since it provides orientation invariance on the account of significantly reduced gait pattern discriminativeness (please refer to Section 4.6 for more details). Univariate signal is also output of uniaxial inertial sensor measuring quantities in single spatial direction. Majority of revised gait recognition approaches rely on multivariate measurements, acquired mostly by triaxial accelerometers either autonomous or combined with triaxial gyroscopes. In current state-of-the-art, multi-sensor inertial measurements were applied mostly for experimental purposes in order to examine the influence of sensor position on the recognition performance. However, these can be also used when the concept of multiple wearable sensors (*i.e.*, body area networks) is applicable as it has been shown that sensor fusion significantly improves recognition efficiency [45,46,47].

### 4.2. Preprocessing

After data acquisition, preprocessing is proposed by some of existing inertial sensor-based recognition approaches. This initial step is intended due to the sufficient preparation of acquired inertial data for further processing, either to remove the noise by applying filtering or to extract shorter segments of inertial data that carry sufficient amount of discriminative information of the user’s gait that are more suitable for efficient processing.

**Table 1:** Specifications of inertial sensors used in representative gait recognition approaches.

	Sensor Model	Sensor Configuration	Number of Sensors	Acceleration (Data for One Sensor)			Gyroscope (Data for One Sensor)		
				Number of Measuring Axes	Range of Measurement	Sampling Rate	Number of Measuring Axes	Range of Measurement	Sampling Rate
*Datasets used in compared approaches*									
Ngo *et al.*, 2014 [27]	ZMP IMUZ, Kionix KXRF9 accelerometer	3 evaluation boards, 1 smartphone Motorola ME860	3, 1	3, 3	±3 g	100 Hz, 100 Hz	3, 0	± 500 ${}^{\circ }$/s	100 Hz
*Approaches*									
Trivino *et al.*, 2010 [48]	Not provided	Stand-alone	1	3	Not provided	10 Hz (constant)			
Ngo *et al.*, 2011 [42]	MicroStrain 3DM-GX3-25	Stand-alone	1	3	Not provided	100 Hz (constant), resampled to 50 Hz	3	Not provided	100 Hz (constant)
H. Sun *et al.*, 2012 [43]	ADXL345	Stand-alone	1	3	Not provided	50 Hz			
Derawi *et al.*, 2013 [49]	Not provided	Smartphone Samsung Nexus S	1	3	±2 g	150 Hz (variable), resampled to 150 Hz using linear interpolation			
Frank *et al.*, 2013 [44]	Not provided	Smartphone HTC Nexus One	1	3	Not provided	28.5 Hz (variable), resampled to 25 Hz using linear interpolation			
Nickel *et al.*, 2013 [50]	ST LIS331DLH	Smartphone Motorola Milestone	1	3	Not provided	127.3 Hz (variable), resampled to 25, 50 and 100 Hz using linear interpolation			
Sama *et al.*, 2013 [51]	ST LIS3LV02DQ	Stand-alone	1	3	Not provided	200 Hz			
Ngo *et al.*, 2014 [28]	ZMP IMUZ, MicroStrain 3DM-GX3-25	Stand-alone	3, 1	3, 3	Not provided	100 Hz, 100 Hz			
Ren *et al.*, 2014 [52]	Not provided	Smartphone HTC EVO	1	3	Not provided	50 Hz			
B. Sun *et al.*, 2014 [53]	Not provided	Smartphone iPhone	1, 1	3	Not provided	Not provided	3	Not provided	Not provided
Zhang *et al.*, 2014 [29]	ADXL330	Wii remote	1	3	±3 g	100 Hz			
Zhong *et al.*, 2014 [45]	Relying on dataset of Ngo *et al.* [27] (experimental) and Frank *et al.* [44] (realistic).								
Hoang *et al.*, 2015 [54]	BMA-150	Smartphone HTC Google Nexus One	1	3	±2 g	27 Hz, resampled using spline interpolation			
Sprager *et al.*, 2015 [46]	Relying on dataset of Ngo *et al.* [27] (experimental) and Frank *et al.* [44] (realistic), resampled to 25 Hz in both cases.								

#### 4.2.1. Filtering

In the context of signal processing, filtering represents one of the crucial steps to perform noise removal. It is also applied by some existing gait recognition approaches, where noise is determined as high-frequency components reflected by various sources (*i.e.*, loose device attachment, misplacement errors, *etc.*). Thus, some approaches apply moving average filter [49,55] or multi-level wavelet decomposition and reconstruction [36,56,57] in order to remove these components. Some approaches also apply zero-normalization in order to remove gravity component from acceleration signals [50,58]. This is applicable if sensor is firmly fixed and aligned with the longitudinal and vertical directions of the global coordinate system. In order to detect rotational offsets, moving average filtering with long windows is also used [37]. Basically, it can be noted that filtering is applied to overcome the limitations due to sensor-induced gait-affecting factors in some manner. However, such approaches turned out to be very limited and non-robust, thus such problem should be tackled in more systematic way (please refer to Section 4.6 for more details).

#### 4.2.2. Gait Detection and Activity Recognition

When observing human movement from a practical point of view, the following alternating phases are reflected in acquired inertial data: movement (gait), quiet stance (including postural sway) and remaining activities, including transitional phases or noise (screen taps, sensor attachment procedure, *etc.*). From the methodological point of view, sections that correspond to gait phases should be identified from the inertial data. Other two phases are not relevant for gait recognition and can be discarded. At this point is it necessary to explain that this procedure is closely related with the problem of activity recognition that can be performed on the basis of inertial data, mostly relying on window-based approaches [59]. In this manner, such activity recognition procedures can be applied as a part of preprocessing step in similar context by inertial sensor-based gait recognition approaches.

Extraction of sections from inertial data that contain gait is by existing gait recognition approaches performed in three different ways: manually, automatically or implicitly as a part of gait cycle detection procedure during the segmentation step. Due to the cyclostationary nature of gait, the latter deserves special attention by employing its positive properties and is presented in Section 4.3.1. However, majority of existing, especially early-phase approaches employed manual segmentation where the primary goal was the evaluation of the approach and applicability in real-world situation was not in the first plan. By manual detection, inertial data is visually inspected and sections that contain gait are annotated easily due to their cyclic property and deterministic shape. On the other side, since the automatic detection of gait sections is essential by approaches that are planned to be applied in real-world, some of the recent approaches leverage basic signal processing or pattern recognition procedures, such as leveraging moving window (*i.e.*, sum of absolute values) [44], classification with decision trees depending on basic features [37] and motion trajectory constraint and signal autocorrelation [27].

### 4.3. Segmentation

Segmentation is a procedure, where acquired inertial data is divided into smaller components that are suitable for further processing. Considering real-world applicability of inertial sensor-based recognition approaches, two important aspects are desired: low computational complexity and low recognition latency. This can be achieved by the approaches that preserve recognition efficiency even when processing very short gait epochs acquired by single walking trial (*i.e.*, few seconds). Segmentation is reasonable especially when considering the cyclostationary property of gait. Thus, inertial data acquired during gait can be divided into gait cycles. One gait cycle is a result of bipedal human movement characteristic and is defined as interval starting from the time instant where one foot makes initial contact with the ground and ending when the same foot contacts the ground again. In contrast with cycle-based approaches, several gait recognition approaches does not apply cycle-based approaches but rather operate directly on short frames with fixed length.

#### 4.3.1. Cycle-Based Approaches

Cycle-detection procedures are the basis for gait speed estimation [9] and can be also applied in the context of gait recognition. Gait cycles are within inertial data reflected as repeating patterns, where one period represents either single gait cycle or single step in a special case when the sensor is attached at the symmetric body position according to the lateral axis (*i.e.*, at center back). Unlike frame-based approaches, cycle-based approaches allow analysis based on individual gait cycles. Aligning procedures also address temporal variations in gait patterns, mainly as perturbations that are consequence of gait-affecting factors (*i.e.*, variations in gait speed). However, it needs to be considered that recognition efficiency directly depends on the performance of gait detection procedure. Besides that, cycle alignment can also generate drastic changes of gait pattern morphology.

Existing cycle-based gait recognition approaches rely on gait detection methods that leverage local extrema analysis in a most common way [23,24,31,32,49,60,61,62,63,64,65,66,67], as well as zero-crossing detection [25,40,41,58,68], salience vector detection [69,70], phase analysis [28,42], detection of dynamic variations [71] and correlation coefficient [36,47,52,56,68]. Followed by gait cycle detection, the length of extracted gait cycles is unified. This can be performed either by length normalization or alignment procedure. Length normalization produces gait cycles of common length, where the gait pattern shape remains untouched. It is performed by interpolation, either by linear [31,49,58,60,63,64,66,67,69,70] or cubic-spline [52]. On the other side, alignment affects the temporal structure of gait pattern aiming to perform optimal matching of two compared cycles. Most of existing approaches that rely on cycle alignment use dynamic time warping (DTW) procedure [32,38,40,41,43,62,65,72], as well as more sophisticated phase-based self-DTW [28,42].

#### 4.3.2. Frame-Based Approaches

Unlike cycle-based segmentation, frame-based approaches do not require any additional processing since acquired inertial data can be framed into either overlapping or non-overlapping segments (frames). These approaches also preserve the morphology of gait patterns. However, frame-based segmentation does not control the information on gait phase as well as does not address temporal variations induced in gait patterns. Many efficient gait recognition approaches rely on frame-based segmentation, where the use of variety of frame lengths was examined. It can be noticed that frame-based gait recognition approaches consider lower-bound determined by a priori knowledge of a lowest expected human gait speed following the assumption that one frame should contain at least one complete gait cycle. Approaches were evaluated with the frame lengths from the following intervals: duration of the longest expected gait cycle (1.4 s [18]) to 3 s [35,44,46,50,51,73,74], 3–5 s [44,46,50,51,72,73] as well as more than 5 s up to 15 s [34,37,46,51,73]. However, some approaches, especially machine-learning-based, experimented with more fragmented segmentation, where segments have length less than the longest expected gait cycle [46,51]. However, complete gait cycles are considered during learning process in such case since all fragments covering one cycle are included in learning set. Furthermore, it should be emphasised that some approaches appeared recently that are able to operate efficiently even without using special segmentation procedure since it is implicitly addressed either by transformation to feature space or recognition procedure [29,46,75].

### 4.4. Transformation to Gait Patterns

A crucial step of each gait recognition approach is to describe gait patterns in a discriminative way allowing for efficient recognition in subsequent step. As already described in Section 3, gait patterns of the individuals are determined by estimation of gait parameters expressed either in physiological manner either implicitly as features. Due to its simplicity and efficiency, the latter turned out to be most convenient way to produce sufficiently discriminative gait patterns. This was also confirmed by the review process which has shown that vast majority of currently available approaches leverage transformation to feature space. Gait patterns represented as features can be obtained in three ways: (a) construction of gait patterns within segmentation step; (b) computation of generic features and (c) advanced feature extraction approaches.

In the first way, gait patterns still hold the explicit information on inertial data. The basis for producing gait templates are either gait cycles or frames obtained by segmentation procedure. These can be passed directly to recognition procedure as unaltered segments [23,40,60,72], either can be processed further. One of the most simple yet efficient approaches is to determine average gait cycle [32,38,39,41,49,52,58,61,68,76,77] extracted from segmented cycles as single representative gait cycles that is used as a template in recognition procedure. In the other way, generic features describe gait patterns by computation of simple parameters, usually in time domain, frequency domain or in terms of statistical parameters. Usually, these generic features can be later within the recognition step used either individually (as a feature vector) or combined (fused on the score level). Approaches that rely on parameters in time domain apply analysis of local extrema [34,36,56,74,78], zero-crossing [55], cycle length [25], gait cycle frequency [36,53,56,78], gait symmetry [53] or dynamic range analysis [53,71]. Concerning parameters in frequency domain, existing approaches leverage FFT coefficients [24,36,56,72,76,78], wavelet transform [65,67], cepstral coefficients [37,50,70,73] and discrete cosine transform [36,50,56,78]. Generic features can be also represented as statistical parameters (mean, values, skewness, kurtosis, higher-order moments) [34,37,55,74,79] and distributions (histograms) [24,34,36,56,78]. Vast majority of recent and also most efficient approaches rely on advanced and sophisticated feature extraction procedures, including PCA-based approaches [80], geometric template matching [33,44], computatuional theory of perceptrons [48], inter-period phase registration [28,42], curve aligning [43], singular spectrum analysis [51], sparse representation of signature points [29], gait dynamic images [45], fuzzy commitment scheme [54] and HOS-based approach [46].

### 4.5. Recognition Procedure

In Section 3 a general principle of biometric recognition is explained. Consequentially, all existing inertial sensor-based gait recognition approaches rely on described two-phased principle including enrolment and recognition phase. In both enrolment and recognition phase each approach uses the same representation of gait patterns in feature space obtained by pre-selected transformation procedure as described in previous subsection. Recognition can be performed in two ways: (a) by pattern similarity matching based on cross-comparison of gait patterns or (b) by machine-learning (ML) approaches where gait recognition is represented as classification problem. In both cases, two groups need to be considered: one group containing enrolled gait patterns, commonly referred as gallery by pattern similarity approaches and learning set by ML approaches, and the other group containing patterns intended for recognition which are commonly referred as probe patterns (pattern similarity approaches) or testing set (ML approaches). By machine learning approaches, each pattern in learning set is labelled by the corresponding class (gait owner). After that, classification procedure assigns one of the labels defined within the learning set to each of the input patterns from the testing set. On the other side, by pattern matching approaches gait patterns are transformed into predefined feature space are compared cross-wise. That means that gait recognition is performed by estimating similarity for any pair comprising a gait pattern of an arbitrary owner from gallery and an arbitrary probe gait pattern.

Approaches that are based on pattern similarity estimation usually rely on simple metrics that measure dissimilarity of compared gait patterns, including histogram similarity [25], Manhattan distance [27,49,71], Euclidean distance [27,40,49,60,61,64,69,76], correlation coefficient [23,24,27,28,46,52,68], Tanimoto distance [27,47] and Hamming distance [54]. Besides simple metrics, the following advanced metrics are introduced: most commonly used DTW or DTW-derived metrics [32,38,42,49,63,64,65], cycle rotation metric (CRM) [62,69], cycle pair matching [63], statistical significance analysis [72] or custom score derived from gait characteristics [48]. Approaches where gait recognition is carried out as a classification problem, rely on commonly used classification techniques, including k-NN (nearest neighbour) [41,44,46,73,79], support vector machines (SVM) [31,33,36,39,43,51,52,58,66,67,78], decision trees [34,74], random forests [44,74], neural networks [34,74,81], hidden Markov model (HMM) classifier [35,50,70], Gaussian mixture model (GMM) classifier [37,45], logistic regression [55], Bayesian network classifiers [74] as well as special classifiers such as classifier for spare-code collection [29].

### 4.6. Consideration of Gait-Affecting Factors

By performing gait analysis, one of the most important goals is to study influence of several factors that directly affect gait. From the standpoint of the inertial sensor-based gait recognition problem, these factors need to be considered to some extent as even small deviations in gait characteristics of an individual can drastically alter gait patterns that are measured as single-point motion trajectories by single inertial sensor unit. Therefore, consideration of gait affecting factors currently represents the greatest challenge in the field of inertial sensor-based gait recognition. First investigations were carried out with the experiments performed in controlled laboratory environment where these factors have not been considered. Recently, when the possibility of gait recognition in realistic and relaxed scenarios was demonstrated, first systematic investigations on gait-affecting factors in this context of gait recognition begin to emerge. Existing approaches address this problem in two ways: (a) by analysing influence of gait-affecting factors, mainly through the recognition performance evaluation or (b) by leveraging new methodological approaches that try to ensure recognition invariance by considering these factors. As described in Section 3, gait-affecting factors can be either: (a) sensor-induced, where the alterations in gait patterns are direct consequence of sensor set-up and (b) consequence of variations in gait characteristics due to physiological or environmental parameters.

#### 4.6.1. Sensor-Induced Factors

Concerning sensor-induced factors, sensor position currently represents the biggest challenge. We must consider the fact that inertial sensors measure single-point motion trajectories that can be significantly different depending on their measurement position as the kinetic and kinematic factors that describe gait are reflected in the acquired inertial data in completely different manner. This results in a production of significantly different gait patterns caused even by small variations in sensor positions on human body. As an example, gait dynamics measured on exposed body locations (*i.e.*, sensor attached at foot) may be reflected as the intensive alterations in inertial data, while by some other more rigid positions during gait (*i.e.*, sensor attached at center back) the variations in inertial data are substantially less intensive. Additionally, the manner in which the sensing devices are placed on the body is also very important. If devices are attached firmly, significant amount of positioning problem is resolved considering the fact that device is placed at the exactly the same spot by each of the following measurements. In practice, this is illusory to expect. Currently all existing approaches rely on the assumption that all measurements are performed by inertial sensor always attached at the same position some also considering small deviations in position as a consequence of the relaxed use by real-world scenarios. Approaches exploit the fact that users commonly prefer some positions more often when carrying sensing devices (*i.e.*, trouser pocket as most commonly used place for storing the smartphone). By doing so, some approaches have assumed fixed attachment of the sensing device achieved by controlled experimental environment (*i.e.*, [27,29]), while the others have considered loose attachment as a consequence of more relaxed use of sensing devices that produce high-frequency oscillations or additional gait pattern variations that are planned to be resolved through filtering or either during enrolment (learning) phase (*i.e.*, [44,52]). Furthermore, the possibility of indirect contact of sensing device with human body during gait is also not overlooked and is covered by some experimental protocols. Namely, users in realistic scenarios can carry sensing devices in a bag, purse, *etc.* [28,42] or even hold them in a hand during gait [74]. Such indirect way of carrying behaves as a low-pass filter and additionally attenuates inertial signal dynamics and consequentially the discriminativeness of gait patterns.

Majority of early inertial sensor-based gait recognition approaches is focused on a inertial data acquired by either fixed or loose attached sensing device on a single pre-determined position permanently used within the context of the approach. Some novel approaches examined the influence of several sensing positions on the the performance of gait recognition either by multi-sensor measurements [27,29] or multiple measurements on different body locations by inertial sensors and tried to show stable recognition performance regardless to the body location to which the inertial sensor is placed. However, all approaches still assume the same position or the same multiple positions covered by multi-sensor fusion for single recognition task. Thus, further advantages concerning sensor positioning are yet to be made. Theoretically, one step further could be supported by collection of gait patterns acquired from large amount of body locations (*i.e.*, similar as by marker-based motion capture [82]) where recognition procedure would be carried out as the problem of inter-subject comparison by enrolled learning sets of that cover gait patterns measured on multiple body locations. In this specific situation the enrolment phase would be rather impractical and exhausting. However, trends show that it could become feasible to some extent in the near future (please refer to Section 7 for more details).

Hand-in-hand with sensor positioning, sensor orientation is another crucial factor that affect gait recognition performance. Regardless to the fact that if exactly the same positioning of sensing device is achieved by each measurement, it is clear that measured gait patterns will be significantly different when altering the initial orientation of the sensing device. One must consider the fact that measured inertial data is fixed to a local coordinate system according to measuring axes of inertial sensor that depend on particular sensor implementation. By measuring inertial data within arbitrary local coordinate system at the same sensor position, corresponding spatial measurements differ and thus cannot be compared directly in the context of gait recognition problem. Between an arbitrary pair taken from the collection of these local coordinate systems there exist a relation, determined by the relative rotation between both corresponding coordinate systems. Thus, the problem of relative sensor orientation is treated in the context of predefined world coordinate system which is usually defined by the longitudinal axis aligned with the forward walking direction, lateral axis aligned with sideways direction the and vertical axis aligned with the earth gravity vector. Thus, existing approaches achieve orientation invariance by considering initial sensor orientation and estimation of relative orientation during movement.

The problem of orientation invariance during gait recognition by inertial sensors has drawn significant attention especially in recent years. Namely, early approaches relied on the use of univariate acceleration signal determined by acceleration magnitude which is invariant to sensor orientation. However, the major problem is that the computation of acceleration resultant from triaxial acceleration signal leads into significant loss of information and significant decrease in discriminativeness of gait patterns. Due to its simplicity, it can also be applied as a supporting step by performing more advanced procedures to deal with the sensor orientation inconsistency [17]. Another approach relies on calibration phase prior to the recognition procedure. Sun *et al.* [53] used data acquired by gyroscope in order to eliminate gravitational component from acquired acceleration data. This approach is based on initial attitude of the sensors and uses quaternions for transformation. Major breakthrough has been made in last two years when more advanced and efficient approaches for resolving the issue of orientation inconsistency by gait recognition problem have appeared. Trung *et al.* [28] proposed efficient optimization-based iterative matching algorithm that simultaneously estimates signal correspondence and relative sensor-rotation between enrolled and verified gait patterns. This approach was further improved [17] by additionally employing gyroscope for sensor tilt correction. Zhong *et al.* [45] proposed invariant gait representation relying on both accelerometer and gyroscope data by introducing gait dynamic images—2D representation that capture invariant motion dynamics over time. Subramanian *et al.* [47] proposed an approach that relies on Kabsch alignment which minimises the RMS error between the rotated versions of the observing and the comparing patterns.

#### 4.6.2. Variations in Gait Characteristics

Majority of existing approaches assume the fact that natural and unaffected gait has been performed during the measurement of inertial data. This is is only partly reasonable when considering the applicability of gait recognition procedure in realistic circumstances since it is known that besides sensor-induced factors, variations in gait characteristics also significantly affect recognition performance. In last few years, new extended datasets were published, where extensive experimental protocols were designed in order to collect data suitable for examination of both physiological and environmental gait-affecting factors on the gait recognition performance [27,83].

Walking speed is a physiological factor that has been by existing inertial sensor-base gait recognition approaches addressed most frequently. This was performed within the segmentation procedure by approaches that adopt cycle alignment. It has been shown that by varying gait speed within acceptable limits gait patterns do not differ much and efficient gait recognition regardless to the walking speed is feasible [52]. Age is another physiological factor that has been examined recently. In [27] it has been shown that especially to the age groups from the both boundaries of the age distribution special attention should be paid due to significantly lower recognition performance. Besides that it has also been shown that there are small variations in the recognition performance based on gender speak in a favour to female subjects [27] resulting with a conclusion that the female’s way of walking is more discriminative. Considering walking directions, majority of existing approaches assumes walking in straight directions, which is sufficient under assumption that recognition should be performed in short gait epochs. However, novel realistic datasets also involve variations in the direction of walk, but systematic investigation of its influence on the recognition performance has not been performed yet. The same could be stated for health-related factors.

Within evaluation procedures of some existing approaches, experimental datasets have also included environmental gait-affecting parameters. The influence of footwear on gait recognition was already examined in early phase [63,64,72] where the recognition accuracy decreased by using shoe types that obstruct the natural gait of walking (*i.e.*, heavy shoes). Gait recognition performance by wearing heavy load (*i.e.*, in the backpack) was also examined [42,72] where it has been discovered that carrying weight changes the intensity of inertial signals. A study of influence of surfaces on recognition performance was examined in [66] where it has been shown that solid surfaces do not have significant influence on recognition performance unlike surfaces that force subject into drastic change of its way of walking (*i.e.*, ice or shallow water). The influence of ground slope on recognition performance was also examined in [27] where it was shown that walking on ascending or descending surface results in a significant decrease of recognition accuracy.

Finally, some approaches rely on data acquired in the wild [44,52], where gait was exposed to variety of physiological and environmental factors. The advantage of such data is to examine whether gait recognition approaches operate in realistic and uncontrolled circumstances, where these factors are implicitly induced in inertial data. Unfortunately, major drawback by such datasets is that all these factors are unlabelled and thus further systematic investigation on these gait-affecting factors is not possible.

### 4.7. Fusion Procedure

As the other conventional biometric approaches, inertial sensor-based gait recognition approaches can also employ fusion procedure. Its role is to combine and consolidate information presented by different sources or modalities [26]. Within presented state-of-the-art, approaches rely on fusion procedures performed on the following levels: sensor-level, feature-level, score-level and decision-level. The use of sensor fusion is very reasonable and commonly used by processing data acquired from inertial sensors. It usually relies on well-known combination of accelerometer and gyroscope data within single IMU or can either employ multi-sensor measurements, where multiple sensors are attached at different locations in order to further improve recognition performance [45,46,47]. However, it should be mentioned that by these approaches, sensor-level fusion is performed implicitly by performing score fusion, where scores are obtained from inertial data collected by individual sensors. Feature-level fusion combines two or more feature sets acquired from inertial data of the same individual and was *i.e.*, used in [46] where cumulants of different orders were fused. Score fusion represents one of the most frequently used fusing procedures in biometric systems due to its simplicity and efficiency. It represents a fusion on a measurement level that combines score output produced by different matchers by recognition procedure. Thus, existing approaches rely on weighted sum of scores [64], SVM-based score fusion [43], product of scores [17], sum of scores [17,46], minimum of score [48], average score [45] *etc*. When only decisions by individual matchers within recognition procedure are produced, decision-level fusion takes its place. Voting scheme [50,53,69,70] is the most frequently used in existing approaches.

## 5. Comparative Analysis of the Representative Approaches

As a result of the third phase of review process, fourteen relevant approaches published in the latest period that provide answers on the research questions provided in Section 2 were compared to the greatest extent. These approaches fully represent current state-of-the-art in terms of methodological procedure these approaches rely on, as well as in terms of relevant performance evaluation. In particular, we were interested in the methodological details, what datasets were used for evaluation procedure, which factors were covered by these datasets and how relevant were the obtained results of the proposed approaches obtained by these datasets in terms of performance and reliability. All representative approaches that were analysed in detail are highlighted in Table 2 sorted by year of publication and alphabetical order of author’s names.

**Table 2:** Methodological details on representative inertial sensor-based gait recognition approaches.

Approach	Sensor Data Used	Preprocessing				Consideration of Gait-Affecting Factors	Methodology	Decision Procedure	Special Remarks
		Filtering and Normalization	Activity (Gait Sequence) Detection	Segmentation	Aligning				
Trivino *et al.*, 2010 [48]	Acc. data in vertical and lateral direction	No filtering, z-score normalization	No	Covered by fusification model	No	No	Computational theory of perceptions	Pattern similarity: score derived from gait characteristics (homogenity, symmetry and the fourth root model)	
Ngo *et al.*, 2011 [42]	Gyr. data (all axes)	No	No	Phase-based cycle detection	Implicitly by time warping function	No	Phase registration supported by linearization of time warping function	Pattern similarity: normalised cumulative DTW score	
H. Sun *et al.*, 2012 [43]	Acc. data (all axes)	Low-pass Butterworth filter at 20 Hz	No	Cycle detection-based	Covered by curve aligning approach	No	Curve aligning	Axis-wise pattern similarity fusion based on DTW, correlation and curve aligning (SVM)	
Derawi *et al.*, 2013 [49]	Magnitude	Weighted moving average	No	Cycle detection: length estimation, peak analysis	Covered by time warping function	Orientation invariance by applying magnitude at the cost of information loss	Average cycle template	Pattern similarity: Manhattan distance (computation on phone side), Euclidean and DTW distance (computation on server side)	Computation on both smartphone and server side
Frank *et al.*, 2013 [44]	Magnitude	No	Sliding window approach (2 s), threshold on sum of absolute values	Fixed-length segments: width of 2 s and 5 s	No	No	Geometric template matching	Classification: random forest, 1-nearest neighbour	First reference for evaluation of recognition in realistic circumstances
Nickel *et al.*, 2013 [50]	Acc. data (all axes, magnitude)	Zero-normalization	No	Fixed-length segments: width of 2 s, 3 s and 4 s	No	No	Mel-frequency and bark-frequency cepstral coefficients	Classification: hidden Markov models, voting	
Sama *et al.*, 2013 [51]	Magnitude	No	No	Fixed-length segments: width of 1 to 10 s	No	No	Signal spectrum analysis (box approximation geometry)	Classification: SVM (Gaussian kernel)	
Ngo *et al.*, 2014 [28]	Acc. data (all axes)	No	No	No	Covered by signal registration	Orientation invariance	Orientation-compensative matching algorithm based on cyclic dynamic programming	Pattern similarity: dissimilarity by the rotation optimization function	First research that sufficiently addresses orientation problem
B. Sun *et al.*, 2014 [53]	Gyr. data (calibration phase), acc. data (recognition phase)	No	No	No	No	No	Gait characteristic parameters (gait frequency, symmetry coefficient, dynamic range, similarity coefficient of characteristic curves)	Weighted voting	Addresses sensor inaccuracies in smartphones
Ren *et al.*, 2014 [52]	Acc. data in vertical direction	No	No	Cycle detection based on a-priori knowledge employing Pearson’s CC	Cubic spline interpolation (300 samples)	Walking speed	Gait cycle template from acceleration trace	Weighted Pearson’s CC (computation on user side), SVM (computation on server side)	Computation on both smartphone and server side, includes placement study and spoofing attack study
Zhang *et al.*, 2014 [29]	Acc. data (all axes)	No	No	Covered by detection of signature points	No	No	Multiple signature points in scale	Classifier for sparse-code collection	
Zhong *et al.*, 2014 [45]	Acc. and gyr. Data (all axes)	No	No	Parameter-wise based on a-priori knowledge	No	Orientation invariance	Gait dynamic images	Cosine distance between i-vectors (GMM-based similarity estimation)	Robust to variations in sensor orientation
Hoang *et al.*, 2015 [54]	Acc. data (all axes)	Wavelet filtering (Db6)	No	Peak detection based on vertical acceleration, all cycles resampled to a fixed length	No	No	Biometric cryptosystem approach (fuzzy commitment scheme)	Hamming distance	Security and privacy preserved system (encrypted gait templates)
Sprager *et al.*, 2015 [46]	Acc. data (all axes, magnitude)	No	No	Fixed-length segment widths based on a-priori knowledge: 0.7 s, 1.4 s, and 2.8 s (experimental); 2.8 s, 4.2 s, 8.4 s and 12.6 s (realistic); variable signal lengths	No	No	Higher-order statistics	Normalized CC	Very short gait epochs, no segmentation or cycle detection needed, variable signal lengths

Trivino *et al.* [48] proposed an approach that is based on computational theory of perceptrons. It used biaxial accelerometer data in vertical and lateral direction that are normalised by z-score normalization. Segmentation procedure was covered by fusification model. Gait recognition was performed by pattern similarity based on score fusion derived from gait characteristics (homogeneity, symmetry and the fourth root model). Ngo *et al.* [42] relied on phase registration-based approach that employs data acquired by triaxial gyroscope, where gait cycles are detected by phase analysis. Phase registration is supported by linearization of time warping function and recognition is performed by using normalized cumulative DTW score. In 2012, H. Sun *et al.* [43] proposed a curve-aligning approach that relied on acceleration data acquired by triaxial accelerometer filtered by low-pass Butterworth filter having cut-off frequency equal to 20 Hz. Cycle detection was covered by curve aligning and recognition was performed by axis-wise pattern similarity score fusion performed by SVM with scores determined by DTW, correlation coefficient and curve aligning. Derawi *et al.* [49] applied average cycle template for gait recognition problem. It originates on the aligned gait cycles obtained from acceleration magnitude obtained by peak analysis-based cycle detection procedure. Recognition procedure was performed on the user side (smartphone) by employing Manhattan distance as similarity metric, as well as on the server side, where DTW and Euclidean distance is used as similarity metric. Frank *et al.* [44] also relied on magnitude of accelerations obtained by smartphone. They proposed an approach based on geometric template matching, where acceleration data was sliced in to fixed-length frames with 2 s and 5 s. Recognition was performed as classification problem by applying random forest and 1-nearest neighbour. Gait recognition approach based on hidden Markov models was proposed by [50] *et al.* By their approach, triaxial accelerometer data was first normalized by zero-normalization and segmented into frames with the fixed length of 2 s, 3 s and 4 s. Cepstral coefficients were extracted as features and recognition procedure was performed as classification problem based on HMM employing voting as decision-level fusion. Sama *et al.* [51] proposed signal spectrum analysis-based approach, where magnitude of acceleration signals were segmented into frames with fixed length from 1 s to 10 s. Gait recognition was performed as classification task by employing SVM with Gaussian kernel. In 2014, Ngo *et al.* [28] proposed new orientation-compensative matching algorithm. It represents first approach that sufficiently and systematically copes with the orientation problem by gait recognition. Orientation compensative matching algorithm is based on cyclic dynamic programming and recognition procedure is performed by dissimilarity estimation of gait patterns based on optimization in rotational space. B. Sun *et al.* [53] introduced initial calibration phase that addresses sensor inaccuracies in smartphones. It relies on both accelerometer and gyroscope data. Recognition procedure was performed by weighted voting based on gait characteristic parameters (gait frequency, symmetry, dynamic range and similarity coefficient of characteristic curves). Another gait cycle-template-based approach was proposed by Ren *et al.* [52], where acceleration data in vertical direction was taken and cycles were detected by procedure relying on Pearson’s correlation coefficient. Cycles were then aligned by cubic interpolation and gait cycle templates were generated. The proposed approach assumed computation on user side, where weighted Pearson’s correlation coefficient was applied for the recognition procedure, as well as on server side, where SVM wass employed for recognition procedure in terms of classification. Another efficient approach have been proposed by Zhang *et al.* [29] which exploited multiple signature points in scale. It relied on triaxial accelerometer data and recognition procedure was performed as classification task by special classifier for sparse-code collection. Zhong *et al.* [45] proposed another orientation invariant gait recognition approach that was based on special transformation of both acceleration and gyroscope data into gait dynamic images. Recognition procedure was performed by GMM-based similarity estimation, employing cosine distance between i-vectors. A new biometric cryptosystem approach was proposed by Hoang *et al.* [54] which included encryption of gait templates. Data acquired by triaxial accelerometer was filtered by using wavelet transform (Db6 mother wavelet) and gait cycles were extracted by detection procedure relying on peak analysis. Gait cycles were exposed to fuzzy commitment scheme and recognition procedure was performed by pattern similarity estimation using Hamming distance. Sprager *et al.* [46] proposed new approach based on higher-order statistics that operates on very short gait epochs with variable lengths. It was evaluated by using triaxial accelerometer data sliced to frames with the lengths varying from 0.7 s to 12.6 s, employing both fixed and variable frame lengths. Recognition procedure was carried out by pattern similarity estimation using normalized correlation coefficient as metric, as well as classification task employing 1-nearest neighbour.

### 5.1. Evaluation Datasets

Proper evaluation datasets are essential when performing evaluation and comparison of gait recognition approaches. These datasets should be broadly in line with the following properties: (a) datasets should include inertial data acquired from the number of subjects as great as possible covering wide population according to the gender and age; (b) inertial data should be acquired by users within laboratory (experimental) environment in controlled conditions as well as in realistic circumstances; (c) measurements should include short-term (lower latency) as well as long-term measurements (gait pattern redundancy) and (d) experimental protocol should cover as many gait-affecting factors as possible (either sensor-based, physiological or environmental) that should be well structured, systematically observed and properly labelled. In order to perform the evaluation in a general and consistent way, the following four crucial factors are introduced based on above mentioned properties: performance, reliability, robustness and efficiency. Performance is a principal factor that provides a quantitative measurement on recognition procedure by several metrics (see Section 5.2 for more details). In our context, reliability can be described as stable recognition performance by repeatable measurements regardless to the number of subjects and measurement trials included in datasets. Robustness reflects the ability of the approach to operate in non-ideal and exceptional circumstances, such as gait-affecting factors, whereas efficiency is related to latency and real-time processing, including ability to perform efficient recognition on short gait epochs as well as computational complexity. In the following, datasets used for the evaluation of representative inertial sensor-based gait recognition approaches are presented, where some of them are publicly accessible and have become reference for a systematic comparison analysis of performance. All details on evaluation sets described in this section are highlighted in Table 3 and Table 4 for all representative approaches.

**Table 3:** Evaluation of representative inertial sensor-based gait recognition approaches (part 1/2).

Approach	Experiment Description					Length of Shortest Gait Epoch Used for Recognition	Validation		Performance		Special Remarks
	Dataset Reference	Type of Validation	No. Subjects (M + F)	Protocol Description	Measurement Length		Gallery Data	Probe Data	Measure	Value	
Ngo *et al.*, 2014 [27]	[27]	Experimental	744 (389 + 355); 495 (IMUZ) and 408 (smartphone)	Two datasets including level- (9 m), up- and down-slope walk (3 m)	Short sequences, acquired by 1 min. long sessions		First level walk	Walk in opposite direction, slope walks	EER	Derawi *et al.* [62]: 14.3%, Rong *et al.* [41]: 14.3%, Gafurov *et al.* [63]: 15.8%, Ngo *et al.* [42] (gyroscope): 20.2%	Largest currently available IMU-based gait dataset, equal distribution of gender and age range
Trivino *et al.*, 2010 [48]	[48]	Experimental	11	Each subject walked 20 trials with self-selected gait speed	10 steps each trial	10 steps	Leave-one-out cross-validation of each trial against the remaining trials		EER	3%	
Ngo *et al.*, 2011 [42]	[42]	Experimental	32 (25 + 7)	Normal walk along an indoor corridor, 5 sequences for each subject carrying bag with weight increased on each trial	2 min long trials (approx. 64 gait periods per trial)	Half of extracted gait cycles from each trial (approx. 1 min)	Half-half validation and leave-one-out validation for each scenario		EER	6%	
H. Sun *et al.*, 2012 [43]	[43]	Experimental	22 (16 + 6)	Four trials for each subjects	20 m long corridor	4 gait patterns	Two-fold cross-validation		EER	0.8% (fusion) 3% (non-fusion)	
Derawi *et al.*, 2013 [49]	[49]	Partly realistic	25	3 trials for each subject with 3 different walking speeds (slow, normal, fast)	30 m long corridor	Whole collection of gait pattern in trial	5 enrolled users, real-time evaluation based on gait of 25 users		Accuracy	89.3%, *p*(FP) = 1.4%	
Frank *et al.*, 2013 [44]	[44]	Realistic	20 (10 + 10)	2 measurements on different day with walking on the same trail on different surfaces, different clothing apply on each day of measurement for some subjects	15 min	2.8 s	Trials measured on first day	Trials measured on second day	Accuracy	42% (TDEBOOST), 63% (applied label smoothing)	First realistic experiment
Nickel *et al.*, 2013 [50]	[50]	Realistic	48	Two phases: enrolment (shorter straight walk), authentication (long walk inside building on predefined route)	10 s (enrollment), longer walk (authentication)	4 s (best result)	Data from enrolment phase	Data from authentication phase	EER	15.8%	
Sama *et al.*, 2013 [51]	[51]	Experimental	20	Walking with normal speed, 2 trials on the same day, sensor reinstalled between measurements	20 m long corridor	7 s (best result)	First trial	Second trial	Accuracy	96.4%	

**Table 4:** Evaluation of representative inertial sensor-based gait recognition approaches (part 2/2).

Approach	Experiment Description					Length of Shortest Gait Epoch Used for Recognition	Validation		Performance		Special Remarks
	Dataset Reference	Type of Validation	No. Subjects (M + F)	Protocol Description	Measurement Length		Gallery Data	Probe Data	Measure	Value	
Ngo *et al.*, 2014 [28]	[28]	Experimental	47 (32 + 15)	16 trials per subject: two days, 2 weights, 4 sensors	Each trial 2 min, about 64 gait periods, 90 m long walking path		Data acquired on first day (by 3DM-GX3-25 sensor)	Data acquired on second day (from all sensors)	EER	10%	
B. Sun *et al.*, 2014 [53]	[53]	Partly realistic	10	Straight walk on two surfaces: pavement and grass, 40 sets of data for each subject	Each trial 10 s (9–10 gait cycles)	10 s	One set of data for one subject	Remaining 3 sets of data	Accuracy	All correct	
Ren *et al.*, 2014 [52]	[52]	Realistic	26	Casual walking of users, 3048 trials in half year, 2 types of trials: short and long; experiment included gait speed variations as well as spoofing scenario (8 adversary and 10 spoofing users)	Long trials: about 10 min; short trials: 10, 20 and 40 s (detection latency, walking speed and placement studies)	20 s for stable accuracy	Several gallery and probe pools for different evaluation phases		Accuracy, FRR	Accuracy over 80% (user-side), over 90% (server side), FP rate under 10%	Includes important studies: step cycle identification, detection latency, walking speed, placement and possibility of spoofing
Zhang *et al.*, 2014 [29]	[29]	Experimental	175 (153 in seasons S1 and S2, 22 in one season S0)	2 recording seasons on level walk, 6 trials per subject in one season, 1 week–0.5 year time interval between two seasons	20 m straight level walk, 7–15 s for single trial (7-14 gait cycles)	7–15 s	Identification: S1 or S2 for enrolment (as well as S0), remaining for identification; authentcation: S1 and S2 into threefolds, multiple targets per fold and probes per target (exhaustive protocol		EER (authentication), accuracy (identification)	95.8% accuracy for identification, 2.2% EER for authentication	Exhaustive evaluation, data acquired from multiple sensors simultaneously
Zhong *et al.*, 2014 [45]	[27,44]	Experimental ([27]), realistic ([44])	*	*	*	Entire signals	*	*	EER (experimental), accuracy (realistic)	Experimental: 6.8% EER (accelerometer), 10.9% EER (gyrometer), 5.6% EER (fused); realistic: 66.3% accuracy	
Hoang *et al.*, 2015 [54]	[78]	Partly realistic	38 (28 + 10)	Acquisition of 16 gait templates, each gait template consists of 4 consecutive gait cycles	At least 64 steps to generate 16 gait templates	8 random gait templates	Half-half random selection of gait templates		EER, FAR, FRR	0%, 16.2%, 3.5%	
Sprager *et al.*, 2015 [46]	[27,44]	Experimental ([27]), realistic ([44])	*	*	*	1.4 s (both experimental cases), 12 s (realistic)	*	*	EER (experimental), accuracy (realistic)	Experimental, single sensor: 10.1% EER, sensor fusion: 5.5% EER; realistic: 69.4% accuracy	Experiment on very short gait epochs, variable epoch length

Number of subjects included in dataset is an important factor by evaluation of the approaches in terms of reliability. Simple approaches in the early period were usually evaluated by using dataset with small number of subjects, approx. 10 subjects and less. It has been shown that by increasing of number of subjects within the dataset, the recognition performance have decreased significantly by these approaches [27]. Thus, majority of existing approaches from last decade are evaluated with the datasets that include inertial gait data from at least 10-30 subjects [43,44,48,49,51,52,53] as well as from 31 up to 50 subjects [28,42,50,54]. Major problem was that distribution of age and gender was not sufficiently addressed and number of subjects was way too low for determining reliability of the approach in terms of its potential for wider application. Thus, larger datasets that included inertial gait data from 100 and up to 200 subjects appeared [29,47,61]. Finally, Ngo *et al.* [27] have published inertial sensor-based gait dataset that have received a particular attention. Not only that the number of subjects included in dataset is significantly larger that all existing datasets since it includes inertial gait data obtained from 744 subjects, but also has almost equal distribution of subjects according to gender and ages and systematic analysis that confirms the influence of the number of subject on the recognition performance in terms of reliability is also performed. Currently, this dataset is largest available gait-based inertial-sensor dataset which has become a principal reference in the field of inertial sensor-based gait recognition.

On the other hand, majority of existing datasets contain inertial data that consider normal gait (without any perturbations) performed in controlled conditions, usually by collected during short walking trials (about few seconds). In order to examine the robustness of gait recognition approaches and to observe the permanence of gait patterns in more realistic and aggravated circumstances, measurements based on more extensive experimental protocols are performed. On the account of the smaller amount of subjects included in the experiments, these protocol rely on longer walking trials (at least few minutes), in naturalistic circumstances that cover casual user activity and as many realistic parameters as possible. Finally, measurement trials are performed in several sessions with longer time intervals between seasons (few days, even months). In this manner, all existing evaluation datasets can be divided into three categories: dataset for experimental purposes, *i.e.*, [27,28,42,43,48,51], partly realistic datasets [49,53,54] that can be considered as almost realistic but in multiple manners do not fully comply to the majority of above mentioned protocl properties, and fully realistic datasets [44,47,50,52]. One of the first and most important datasets was published by Frank *et al.* [44] that includes casual 15 min-long walking trials of 20 subjects on the same trail in two different days. Their dataset represents a reference for the evaluation of approaches in realistic and aggravated circumstances which included casual smartphone placement, walking on different slopes, surfaces as well as different clothing and footwear on different measuring seasons for particular subjects. Nickel *et al.* [50] proposed a dataset constructed by interesting experimental protocol that covered walking trials of 48 subjects on the long predefined trail inside building, including different slopes, surfaces and opening/closing the doors. Ren 2014 *et al.* [52] used dataset that contains 3048 walking trials of 26 subjects that included gait speed variations where duration of single trial was up to 10 min. Subramanian *et al.* [47] have constructed dataset with 101 subjects that included casual wearing of smartphone including variations in phone placement as well as several realistic actions concerning the use of smartphone (talking, writing messages, *etc.*).

**Figure 3:**
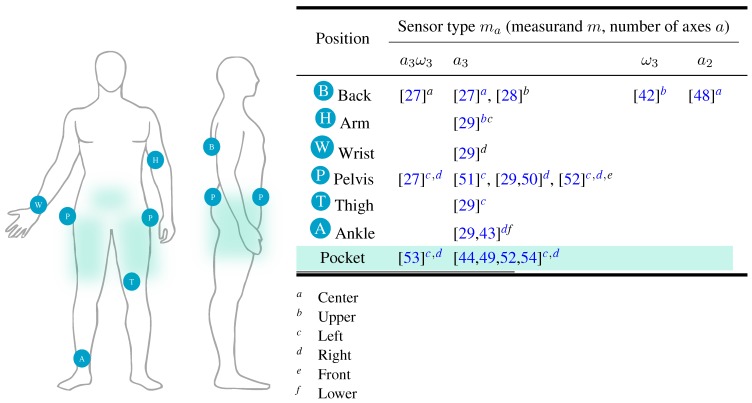
Sensor positions.

Most realistic inertial-based gait datasets cover naturalistic behaviour of subjects during gait involving perturbations in gait patterns due to several gait-affecting factors. Such data collected in the wild is interesting for direct comparison of gait recognition approaches especially in terms of robustness but, however, do not provide insight on particular influencing factors since its contributions are not labelled and are somehow concealed within inertial data. In order to avoid these limitations to a certain extent, experimental datasets should be constructed by employing systematic experimental protocol design, where gait-affecting factors should be properly measured and labelled. In this manner, such dataset could be used for evaluation and further improvement of the existing gait recognition approaches in terms of robustness, *i.e.*, by determining if applications are able to ensure permanence of gait patterns even due to perturbations in gait as a consequence of several gait-affecting factors. However, simultaneous analysis of these factors as well as their coexistent effect on gait patterns is very hard to be performed in a systematic way. Thus, existing datasets were usually constructed by individual observation of one or more particular factors. As already described, the most influential sensor-induced factor is positioning of inertial sensing device during gait. Figure 3 shows typical attachment positions of inertial sensing devices in recent representative datasets. Circles determine sensing positions that were used by experimental protocols that assumed controlled measuring conditions with these sensors usually firmly attached. Particular attention was also devoted to the potential placement positions that are used most frequently and where placement was performed in completely casual manner (represented as shaded area in Figure 3). In this context, some datasets also include multi-sensor measurements. Largest available dataset by Ngo *et al.* [27] along by inertial data measured during gait of 744 subjects with inertial sensor attached to the back also contains inertial data that was collected by inertial sensors attached to left hip, right hip and center back. Furthermore, Zhang *et al.* [29] presented dataset that contains data simultaneously collected by largest amount of inertial sensors so far, from five body locations. The examination of orientation invariance of gait recognition approaches is another important aspect which is usually determined indirectly through the performance evaluation. Thus, Ngo *et al.* [28] constructed a dataset obtained 47 subjects that included multi-sensor set-up, where sensing devices were attached to the equal positions in order to estimate the efficiency of rotational invariance in direct and more systematic way. Besides sensor-induced gait-affecting factors, the following environmental and physiological factors were considered by datasets used for evaluation of the most recent representative approaches: walking speed [48,49,52], surface [53], slope [27] and carrying load [28,42].

### 5.2. Performance Evaluation

In this section the performance of the current state-of-the-art in the area of inertial sensor-based gait recognition are presented by representative approaches with highlights also summarised in Table 3 and Table 4. Performance, especially in terms of reliability and robustness, can be reflected in the light of most commonly used performance measures [26]. These can be divided in two groups, whether the gait recognition is performed as the problem of authentication or identification.

Considering the fact that authentication is usually based on pattern similarity measure which considers predefined authentication threshold, false rejection rate (FRR) can be defined as the portion of genuine recognition attempts that were rejected (with similarity score above and equal to threshold). False acceptance rate (FAR) can be defined as the portion of imposting recognition attempts that were accepted (with similarity score below threshold). Based on these two metrics, equal error rate (EER) represents the most commonly used measure for authentication performance and is determined by rate at which FRR and FAR are equal. By identification problem, the following outcomes can be provided by recognition approach from the user’s point of view: true positive match (TP) where user was identified correctly; false positive match (FP) where user is not enrolled in database and its gait pattern is nevertheless matched with the gait pattern of one of the enrolled users; false negative match (FN) where gait pattern of the user is already enrolled in the dataset but the approach does not return correct identity. Accuracy (usually rank-1 accuracy) is one of the most used measures in this context and is determined as a proportion of all recognition attempts where subjects were identified correctly (TP identification rate).

By measuring performance relying on experimental datasets, approaches were evaluated by data usually divided in two portions (gallery/learning set and probe/testing set)—The partition procedure is explained for each particular approach. The performance of the representative approaches is provided in three groups according to what dataset was used for their evaluation: (a) small dataset containing gait data collected in controlled environment; (b) large dataset; (c) realistic datasets with data collected in the wild. Many of the recent and most efficient approaches are evaluated on both large dataset as well as dataset in the wild [45,46,47].

Concerning approaches evaluated by small datasets, Trivino *et al.* [48] adopted leave-one-out cross-validation of each trial against the remaining trials collected by 11 subjects (20 trials per subject) where evaluation resulted in EER of 3%. Ngo *et al.* [42] relied on both half-half as well as leave-one-out cross-validation for each scenario that included carrying bags with different weights by 32 subjects (5 trials per subject) by recognition performed on gyroscope data. They reported EER of 6%. Sun *et al.* [43] performed two-fold cross-validation on data acquired by 22 subjects with 4 trials per subject and acquired EER of 0.8% when employing fusion procedure and 3% by non-fused recognition. Derawi *et al.* [49] have used dataset that involved gait data of 25 subjects where each subject performed 3 walking trials. Approach was tested by enrolling 5 users while the other served as impostors. They reported accuracy of 89.3% and the probability of FP detection equal to 1.4%. An important contribution of their approach was an ability to perform gait recognition in real-time. Ngo *et al.* [28] reported 10% EER by evaluating their approach on a dataset with gait data collected by 47 subjects measured in 16 trials per subjects within two days (gallery and probe data) with two different weights and 4 different sensors. Hoang *et al.* [54] relied on gait templates that consist of 4 consecutive gait cycles collected from 38 subjects and the approach was validated by half-half random selection of gait templates. They have reported EER of 3.5%. Sama *et al.* [51] employed dataset with gait data of 20 subjects where two trials were collected for each subject, one serving as learning data and the other as testing data. They reported accuracy of 96.4%. Sun *et al.* [53] obtained perfect accuracy when evaluating their approach by gait inertial data of 10 subjects with 40 sets of data for each subjects, where one set was used for learning and the remaining set for testing.

All approaches evaluated by small datasets resulted in rather good performance. However, by presenting largest inertial dataset, Ngo *et al.* reported significant decrease in performance by evaluating some of existing approaches with their dataset, where the following results were reported: Derawi *et al.* [62] 14.3% EER, Rong *et al.* [41] 14.3% EER, Gafurov *et al.* [63] 15.8% EER and Ngo *et al.* [42] (gyroscope): 20.2% EER. This dataset also served as a principal reference exploited by the most recent approaches showing significant improvement in terms of performance, including Zhong *et al.* [45] reporting 6.8% EER (accelerometer data), 10.9% EER (gyroscope data), 5.6% EER (sensor fusion), Sprager *et al.* [46] where EER of 10.1% (accelerometer data) and EER of 5.5% (sensor fusion) were reported as well as Subramanian *et al.* [47] reporting 6.6% EER (accelerometer data), 8.7% EER (gyroscope data) and 6.3% EER (sensor fusion). Besides that, Zhang *et al.* [29] performed both identification and authentication task by using their dataset of gait inertial data collected by 175 subjects. They achieved 95.8% accuracy for the identification task and 2.2% EER when performing authentication.

Considering gait recognition in aggravated and realistic circumstances, dataset provided by Frank *et al.* [44] described in previous subsection served as a reference for the most recent gait recognition approaches. By evaluating their own approach based on geometric template matching, they have reported accuracy of 42% by leveraging TDEBOOST algorithm for classification further improved to 63% when label smoothing was additionally applied. On the same dataset, the following accuracies were reported: 66.3% by Zhong *et al.* [45], 67.5% by Subramanian *et al.* [47] and 69.4% by Sprager *et al.* [46]. Additional realistic datasets used for evaluation of gait recognition approach should be also considered. Nickel *et al.* [50] reported EER of 15.8% in best case scenario for their HMM-based approach. Last but not least, Ren *et al.* reported accuracy over 80% (with computation performed on user-side) and false positive rate below 10% (with computation performed on server-side).

From the standpoint of efficiency and real-time operability, it is desirable that gait recognition should be performed with a minimal amount of user interaction. This is reflected through the smallest expected length of gait epoch that should be provided to gait recognition system by user. Smaller amount of inertial data collected and processed lead into additional decrease of computational complexity and power consumption. Specifically, an open question is at least how many steps an user should perform in a single walking trial provided to recognition system that recognition performance is still preserved. By recent representative approaches, authors relied on different lower bounds determined either by expected gait epoch lengths or expected number of steps in a epoch. We refer to the approaches that have been validated on single short epochs in the light of real-time operability, where only single walking trials in a reasonable limits (about few seconds) were considered for recognition task. Trivino *et al.* [48] experimented with single gait epochs where subjects had to make 10 steps. Derawi *et al.* [49] and Sama *et al.* [51] relied on gait epochs acquired during walk on 30 m and 20 m, respectively. Ren *et al.* [52] reported that the length of gait epochs of 20 s is enough for stable accuracy. The approaches that are capable to operate on shorter single gait epochs, with the length of 10 s and 7–15 s, were proposed by Sun *et al.* [53] and Zhang *et al.* [29], respectively. Finally, it is very interesting that all recent approaches [45,46,47] achieved superior results by employing single gait epochs with the length of approx 5 s. It means that at least 3.5 steps should be provided to recognition system in practice when considering lower bound on expected gait frequency [18].

## 6. Analysis of Impacts

In this section, impacts provided by recent approaches in the field of inertial sensor-based gait recognition are discussed in the light of research questions listed in Section 2. These are provided through the following properties that address the advisability of the practical use of gait recognition as a biometric trait [26]: performance, uniqueness and permanence of gait patterns, collectability, universality and applicability in practice as well as security implications.

### 6.1. Performance

As stated in Section 5, evaluation through performance metrics is the most appropriate and commonly used quantitative measure that also reflect other properties by observing several parameters that cover initially stated research questions. In order to get insight into current situation, performance measures of reliable approaches that have made the most significant contribution by showing good performance in several scenarios are summarised: by evaluation on large datasets including large number of subjects involved in measurements by performing short walking epochs as well as on a realistic datasets containing long walking trials where several aggravating factors are included. Best results achieved by methods in these conditions [29,45,46,47] show EER of approx. 6.5% when evaluated by large datasets (several hundred people) employing accelerometer data only. When fused by other modalities (*i.e.*, gyroscope), EER further decreased to 5.5%. In [29] authors have reported EER of 2.2% evaluated on dataset with 175 people. The number of subjects in this case is still relatively large but significantly lower as in [27]. For fairer comparison would be interesting to evaluate their approach by using same dataset as used by the other mentioned approaches. However, it should be also mentioned that in all these cases approaches have relied on the short walking epochs, where a few steps was sufficient for efficient recognition.

On the other side, performance measures of gait recognition evaluated in the wild, where several uncontrolled factors have affected gait patterns of the subjects, drop significantly. Representative approaches [45,46,47] evaluated by dataset proposed by Frank *et al.* [44] have shown accuracy under 70%. The problem is that the factors that cause perturbation of gait patterns are not considered specifically as they are implicitly involved in both enrolled and testing sets of inertial data used in recognition procedure. Therefore it can be stated that currently there is a lack of approaches that would address the gait permanence in more comprehensive way (see the following subsection for more details). Nevertheless, it can be noticed that by the recognition attempts that involve realistic datasets where these factors are not prominent, accuracy around 90% and more has been reported [29,49,52].

In order to give a better sense and properly situate the performance of inertial sensor-based gait recognition in a global manner and side-by-side with more conventional recognition systems, it should be mentioned that reported performance metrics are fully comparable *i.e.*, with the results of the latest state-of-the-art in the field of face recognition in realistic circumstances [84,85]. It must be also considered that face recognition is currently one of the most studied biometric traits which has been in contrast to the inertial sensor-based gait recognition present for a substantially longer time. However, by comparing gait recognition in the light other biometric traits, the possibility of their combination can be conveyed in terms of biometric fusion but to the author’s knowledge, inertial sensor-based gait recognition has been not yet considered in this context, thus leaving this problem to further research activities.

### 6.2. Uniqueness and Permanence of Gait Patterns

Uniqueness represents a foundation for successful recognition and reveals whether gait pattern is sufficiently different across individuals, but very similar for particular individual on the other side. Uniqueness of inertial sensor-based gait recognition has been reflected within the existing state-of-the-art by efficient performance in terms of low values of FAR obtained by evaluation performed on large amount of subjects in controlled conditions. However, gait-affecting factors can significantly affect discriminativeness of gait patterns according to the individuals. This is directly related to the permanence of gait patterns and can be thus addressed in the similar context. Preservation of gait pattern permanence is the biggest challenge when collecting inertial data in uncontrolled conditions. Sensor-induced part of gait-affecting factors have been well addressed recently by introducing new approaches that overcome orientation inconsistency as well as some multi-sensor-based investigations that have explained the influence of sensor position considering the assumption that sensing device should be stored at the same, frequently used positions during gait. However, environmental and especially physiological gait-affecting factors were investigated and resolved poorly—Performance metrics decrease significantly since the permanence of gait patterns is not reached. In such case, most efficient approaches are usually prone to higher FRR. By further systematic investigation and appearance of more comprehensive approaches, permanence of gait patterns could be reached more or less efficiently. Thus, gait recognition approaches in current situation demand natural gait which is reasonable and completely applicable if there are no sudden drastic deviations in gait characteristics. Consequentially, gait pattern permanence is the most sensitive factor that concerns all existing inertial sensor-based gait recognition approaches.

### 6.3. Collectability

Collectability is one of the factors that speaks in the favour of the inertial sensor-based gait recognition approaches in comparison with other biometric traits. Considering the fact that the proposed approaches are aimed to smartphone and tablet users, inertial data is acquired simply through the mobile applications installed on these devices. As indicated in the Introduction, there are two limitations that should be addressed in this context: power consumption and data connectivity. It has been shown within the state-of-the-art that some of the most reliable approaches [46] are able to efficiently operate on inertial data acquired by low sampling frequencies (about 25 Hz) as well as on short (interrupted) frames of gait—Both corresponding to above mentioned limitations. Furthermore, with the development of technology, battery life of smart devices is increasing, as well as permanency of their connectivity is raising significantly. Therefore, the paradigm of IoT and cloud computing are breaking forth rapidly also in this context. As already indicated and evaluated by some existing approaches, gait recognition can be processed in two different ways: either on user-side, where computation is performed directly on sensing device or either on server-side. While performing recognition on the user-side, computational complexity is usually reduced on the account of decreased performance [49,52]. In the second way, the role of smart devices, supported by mobile applications, is simply to collect and to send raw inertial data to the cloud infrastructure where the enrolment or recognition phase is performed. Thus, advanced and more computational complex approaches can be performed by powerful computational capacities on the server side that provide light-weight data feedback to the user-side after the recognition procedure is completed.

### 6.4. Applicability in Practice

Universality reveals if every person possesses the biometric trait on which relies the proposed recognition approach. There are two conditions that need to be met for the users in order to include subject into biometric system: permanent walking ability and the possession of the device capable to measure inertial data. According to recent disability reports (as stated in Summary Health Statistics for U.S. Adults: National Health Interview Survey, 2012, Tables 18 and 19) about 92.7% of adults from U.S. that are 18 years and older is able to walk a quarter mile and more. We can assume this portion of population as the potential candidates for the proposed biometric approach. Furthermore, considering the portion of population that own a tablet or smartphone with integrated inertial sensors based on the In-Stat report referred in Section 1 it can be concluded that inertial sensor-based gait recognition is partially universal. However, since the appearance and the use of smart devices is emerging, it is reasonable to assume that the portion of potential users will increase significantly in the near future.

From the user’s point of view, mobile applications applied to process inertial data acquired during movement (*i.e.*, activity monitoring, fitness and health-related mobile applications) are becoming extremely popular recently. Thus, it can be concluded that the upcoming period is appropriate for moderate introduction of the security and biometric mechanisms in the same context. Namely, all existing mobile applications rely on the same principle of data collection. It is expected that the perception of gait as a biometric trait relying on inertial sensors would be positive, mostly due to the fact, that such approach is analogous to the above mentioned and well accepted mobile applications in terms of data collection. Another important aspect that speaks in a favour of the usability of the inertial sensor-based gait recognition approaches is passive cooperation of the users during the recognition procedure where no special user interaction is needed except walking. In comparison with other biometric approaches (*i.e.*, fingerprint or iris recognition) that require special sensors and active cooperation of the user in order to collect data in a proper way (*i.e.*, fingerprint or iris scan), presented approaches rely on the sensors and devices that users possess and are also willing to use them frequently.

### 6.5. Security Implications

Security is one of the most important aspects by applying inertial sensors for measuring gait as a biometric trait. Therefore, the possibility of circumvention has to be examined. In this context, the following three possible attacks and countermeasures at the user level could be performed: spoofing, impersonation/mimicking and obfuscation/avoidance. Considering classical spoofing, it has been shown that faking gait as a biometric trait is more difficult than by other known biometric systems (*i.e.*, fingerprint-fake finger, voice recognition - recorded voice, *etc.*) since gait is represented as a dynamic trait (it requires several inertial data samples acquired in a time frame) which is also difficult for direct human perception, as well as raw inertial data is difficult to interpret at the first glance. From this point of view, the only option would be to collect one of appropriate motion profiles of users stored in database and perform corresponding mechanical movement of the device in some way. Thus, circumvention of inertial sensor-based gait authentication approaches by impersonation is more likely and easier to perform. There has been already some interesting investigations performed right on this topic. Gafurov *et al.* [61] have reported that a minimal effort impersonation attack does not significantly increase the chances of impostors being accepted, but the attacker with the knowledge of the closest math can be serious threat to the system. In this specific case, it needs to be explained that one of the earliest approaches with significantly higher EER was used. Same findings were further revealed by [86] arguing that gait impersonation is very difficult task and subject’s physiological gait characteristics work against it when one wants to imitate someone else’s walking. Therefore, the anticipation that at least similar assumptions also hold for more efficient approaches is reasonable. This was recently proved by Ren *et al.* [52] where robustness to random and mimic attacks under different sensor placements was achieved resulting in a comparable performances of the approach after the evaluation by either of the hostile scenarios. However, obfuscation is a major drawback of such biometric trait. As already indicated when considering the problem of gait permanence, only natural gait pattern for each user is permanent as biometric trait, including the deviations due to the gait-affecting factors. The problem is that the user can consciously influence on the gait pattern (*i.e.*, drastically or even randomly changing the way of his/her walking) if he does not want to be identified or authenticated.

## 7. Conclusions and Outlook

**Figure 4:**
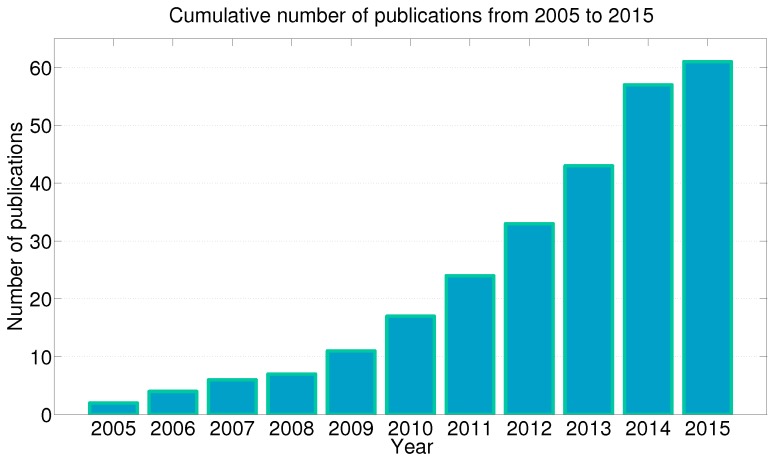
Number of papers in the area of inertial sensor-based gait recognition published in last decade (based on the references considered in review process).

Most recent publications in the field of inertial sensor-based gait recognition indicate that research activities in this field are on the rise. This can be confirmed by Figure 4 which shows cumulative number of publications in last decade where all correspond to the references used in this review paper (please note that for 2015 only the first half of year is considered). It can be concluded that inclination in the number of papers is tightly connected with the popularity and wide usability of inertial sensors, especially as a significant part of smart devices. These ubiquitous devices hand-in-hand with the willingness of the users for their frequent use is a strong foundation for the wide applicability of gait recognition in the practical purposes, implemented either as a stand-alone solution, an additional step by conventional identification or authentication mechanisms or even through the fusion by other biometric approaches in more complex security and surveillance systems.

Currently, first transition attempts of existing approaches from the laboratory to the real-world applicability are in the progress, concluded by the appearance of first prototype systems presented to a broader audience. This has been encouraged by good performance in terms of reliability, robustness and efficiency of some most recent gait recognition approaches that operate well in real-world circumstances. To the author’s knowledge, there exist some prototype solutions, featuring cloud-based service based on the approach published in [46] presented at several demonstrations and workshops in business environment where it has been well-accepted by wider audience. However, in order to achieve inertial sensor-based gait recognition that is enough reliable, robust and efficient to be resulted in wide applicability and commercial availability, new comprehensive approaches will have to be designed following the directions that can be discerned on the basis of current state-of-the-art presented in this paper. These have to cover a plenty of open issues and limitations that should be addressed in following research activities. We have already declared that gait represents rather unstable cyclostationary process that can be perturbed by many gait-affecting factors. Thus major effort will be made in terms of adequately addressing these factors. Influence of these factors should be analysed thoroughly and systematically in a separate way or by investigation of factor inter-influence behaviour of the individual’s gait, also caused by factors that have been unjustifiably neglected to this time (*i.e.*, health-related factors). Thus, new open and extensive datasets designed by protocols that include variety of factors that directly or indirectly influence gait have to be constructed and made available to research groups dealing with the problem of gait recognition in order to achieve further breakthrough by proposing novel comprehensive gait recognition approaches evaluated and fairly compared by using these datasets. In any case, trends show that concepts of IoT and big data analysis will be soon indispensable in this context as smart devices are nowadays well-integrated into global network, usually leveraging the concept of cloud computing. Variety of short-term data from several sensors as well as user actions is collected and can be stored and further processed in the cloud. Thus, gait recognition problem could be also adopted in this context. This could lead to some new accomplishments, *i.e.*, high reliability of enrolled gait patterns could be achieved based on data redundancy. Also, the problem of sensor position during the gait measurement which currently represents the biggest limitation could be addressed to a certain extent. Additionally, by appearance of new sensing devices which hold the potential to become widely and frequently used in combination with smartphones, *i.e.*, smart watches, multi-sensor fusion approaches could further improve recognition performance.

Finally, we can conclude that inertial sensor-based gait recognition represents an emerging and attractive field of research that will be definitely receive further attention in the forthcoming period, especially by examining the possibilities to cope with remaining limitations and open problems. Achievements induced by current state-of-the-art confirm that it holds great potential for further development, especially in terms of applicability and wide usability in the near future. We hope that this paper will encourage further thorough investigations performed by the experts from the area of sensors, pattern recognition, biometrics and other related communities.
